# Ferulic Acid and Polyferulic Acid in Polymers: Synthesis, Properties, and Applications

**DOI:** 10.3390/polym17202788

**Published:** 2025-10-17

**Authors:** Mateusz Leszczyński, Mariusz Ł. Mamiński, Paweł G. Parzuchowski

**Affiliations:** 1 Institute of Wood Sciences and Furniture, Warsaw University of Life Sciences, Building No. 34, 159 Nowoursynowska St., 02-776 Warsaw, Poland; mariusz_maminski@sggw.edu.pl; 2 Faculty of Chemistry, Warsaw University of Technology, 3 Noakowskiego St., 00-664 Warsaw, Poland; pawel.parzuchowski@pw.edu.pl

**Keywords:** bio-based polymers, biomaterials, ferulic acid, functional polymers

## Abstract

Ferulic acid (FA), together with its polymers and derivatives, has been attracting growing attention as a building block for advanced sustainable polymeric materials due to its renewable origin, intrinsic antioxidant activity, and potential for biodegradability. This review aims to provide a comprehensive overview of recent progress in the synthesis and functionalization of FA-based polymers, covering polymerization strategies, enzymatic modifications, and grafting approaches. The physicochemical characteristics of these materials are discussed, with particular emphasis on thermal stability, antioxidant performance, controlled release of active agents, and their impact on the mechanical and barrier properties of polymer matrices. Furthermore, key application domains—including biomedicine, food packaging, and environmental engineering—are examined, highlighting both the advantages and current limitations associated with FA utilization. Finally, perspectives are outlined regarding the necessity for further research to enhance bioavailability, stability, and synthetic efficiency, as well as the potential of FA-derived polymers in the development of next-generation, functional, and environmentally sustainable materials.

## 1. Introduction

Interest in the scientific and technological field related to polymers has increased significantly in recent decades, contributing to the dynamic progress made in plastics and polymer materials. During the 20th century, the polymer industry was heavily dependent on petroleum-based chemistry, refinery processes, and related technologies. The boom of the petrochemical industry in the second half of this century was of fundamental importance for the development of chemical sciences, leading to exceptional growth in the sector of organic chemistry.

Despite growing controversies regarding the negative environmental impact of fossil fuel-based processes, the industrial sector resisted modifying raw materials and technologies until crisis conditions arose [1]. During this period, numerous discoveries were made concerning new monomers used in polymerization reactions, which enabled synthetic materials derived from renewable resources to gain an increasing position in the global market. Thanks to their wide application in the production of everyday goods—such as polylactide (PLA) used in packaging [2], multilayer films made from PLA and soy protein for biodegradable packaging [3], and thermoplastic starch (TPS) biocomposites reinforced with nanocellulose for food packaging applications [4]—these materials have attracted significant attention.

Scientific progress at the turn of the 19th and 20th centuries led to the systematic replacement of natural polymers (e.g., casein-based adhesives used in aircraft plywood production [5]) by their synthetic counterparts. This phenomenon was mainly economically driven—the cost of petroleum-derived raw materials was significantly lower than the cost of processing biopolymers or natural fibers. Although renewable-based polymeric materials remained present on the market, their significance was marginal due to limited investments compared to the substantial funds directed into the petrochemical sector [6].

Currently, the polymer industry faces two fundamental barriers: the principles of green chemistry to be met [7] and the gradual depletion of non-renewable resources. A potential solution may be the intensification of research into the use of renewable raw materials as an alternative to fossil resources, but more importantly the commercialization of new sustainable solutions. Unlike fossil-based resources, renewable sources are widely available, making them strategically attractive from the perspective of global raw material security and environmental stewardship [8].

Bio-based polymers are derived from renewable resources, including plants (e.g., starch, lignin, soy protein) and microorganisms (e.g., pullulan, polyhydroxyalkanoates (PHA), as well as agricultural and forestry by-products, which constitutes a significant step toward the development of sustainable materials [9,10]. They can be obtained from these resources through chemical, physicochemical, or biochemical transformations. Thanks to the renewable origin of their components, bio-based polymers offer numerous advantages, such as a lower carbon footprint and increased biodegradability [11,12].

Although it might be assumed that materials produced from renewable and often biodegradable sources will also be biodegradable, this feature is not guaranteed. The chemical structure, type of functional groups, degree of crosslinking, or the presence of non-biodegradable segments may result in materials that do not exhibit the expected biodegradability [13,14], e.g., bio-ethylene (Bio-PE) or bio-polypropylene (Bio-PP) [15].

Due to the frequent parallel use of the terms “biodegradable” and “bio-based” in the scientific literature, it is necessary to distinguish between these two types of polymers. A biodegradable polymer is one that can undergo breakdown and degradation due to biological agents. Biodegradation, regardless of the origin of the material (biological or synthetic), is the process of chemical decomposition of a substance into compounds harmless to the environment [16]—optimally CO_2_ and water.

This process begins with the fragmentation of high-molecular-weight polymers into shorter, lower-molecular-weight chains [17,18], after which further degradation depends on the activity of microorganisms. Depending on environmental conditions—whether aerobic or anaerobic—microorganisms transform degradation products mainly into carbon dioxide, water, and methane [19].

According to the definition of the International Union of Pure and Applied Chemistry (IUPAC), bio-based polymers are those that are wholly or partly derived from biological products obtained from biomass, including plant, animal, marine, or forestry materials [20].

The most important advantage of bio-based polymers is the possibility of replacing non-renewable resources with biologically derived raw materials, which is a key step toward sustainable production and the development of a low-emission plastics industry with a reduced carbon footprint. An additional benefit is that biodegradable bio-based polymers account for about one quarter of the total number of bio-based polymers (depending on environmental conditions). Next-generation polymers can significantly contribute to reducing dependence on fossil resources and at the same time provide a potential solution to the problem of plastic waste accumulating in the environment, which is neither collected nor processed [21,22].

One of the potential natural-origin raw materials, yet still underappreciated, is FA (3-methoxy-4-hydroxycinnamic acid). An important source of FA, due to its high content, is lignocellulosic biomass. This compound occurs mainly in the form of esters bound to arabinoxylans in plant cell walls. Particularly rich sources include plant residues generated in agriculture and the food industry, such as corn stalks, bagasse (a by-product of sugarcane processing), wheat, barley, and rice straw, as well as residues from sugar beet processing. The high content of FA in such materials makes them valuable feedstocks for its industrial-scale recovery using chemical, enzymatic, or microbiological methods [23,24].

It is distinguished by its antioxidant activity and ability to absorb ultraviolet radiation, making it a valuable component for the design of functional polymeric materials intended for specialized applications, such as films, cosmetic materials, or plastics used in medicine [25,26,27]. Nevertheless, FA is characterized by limited thermal stability during processing and relatively slow kinetics of reactions with free radicals [28].

Oligomers and polymers synthesized from FA can be produced by various techniques, such as enzymatic polymerization, anionic polymerization, and controlled radical polymerization [29,30,31]. Polymers derived from FA contain reactive phenolic and carboxylic groups, enabling further functionalization and control of their thermomechanical properties [32]. These materials can be modified to obtain hydrophilic properties, pH-responsive behavior allowing for controlled FA release, as well as enhanced antioxidant and antibacterial activity [25,26,27,33].

**Figure 1:**
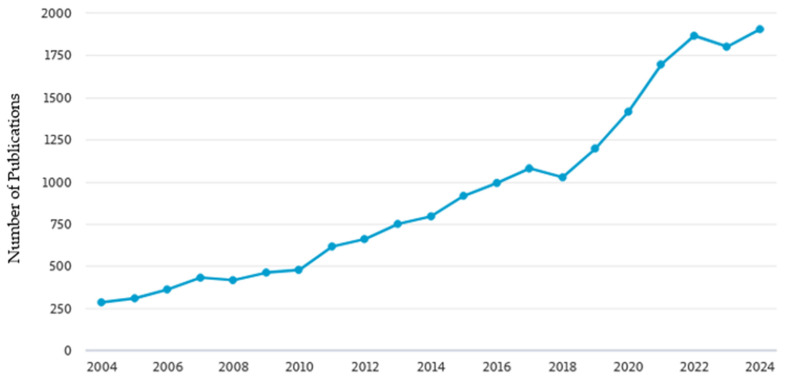
Number of scientific publications retrieved from the Scopus database using the query TITLE-ABS-KEY (“ferulic acid”) AND PUBYEAR > 2003 AND PUBYEAR < 2025, limited to research articles and reviews (https://www.elsevier.com/products/scopus (accessed on 11 August 2025)).

**Figure 2:**
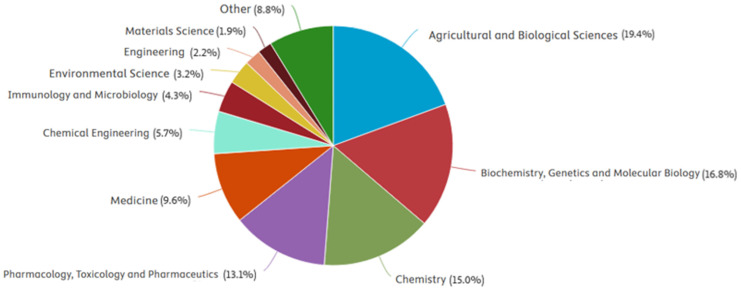
Distribution of scientific publications retrieved from the Scopus database using the query TITLE-ABS-KEY (“ferulic acid”) AND PUBYEAR > 2003 AND PUBYEAR < 2025, limited to research articles and reviews (https://www.elsevier.com/products/scopus (accessed on 11 August 2025)).

In recent years, growing interest has been observed in the application of FA and its derivatives in materials engineering, particularly in the field of polymers and biomaterials. This trend is confirmed by bibliometric data from the Scopus database, showing a systematic increase in the number of publications concerning FA in the years 2004–2024 (Figure 1). Such growth indicates a rising awareness of the potential of this substance in the design of innovative polymeric, biomedical, and packaging materials. The thematic distribution of these publications is presented in Figure 2, illustrating the main research areas in which ferulic acid has been most frequently investigated

FA can serve as a crosslinking agent, functional additive, or nanocomposite component, positively affecting the mechanical properties, durability, and biological activity of polymeric materials [34,35]. Its derivatives, such as polyferulans, are being investigated for applications in biomedicine, biodegradable materials, food packaging, and controlled drug release systems [34,35,36].

The aim of this review article is to present the current state of knowledge on the applications of FA and polyferulic acid in polymers, with particular emphasis on their properties, mechanisms of action, and potential directions of development in the context of modern functional materials.

## 2. Chemical Characteristics and Sources of Raw Materials

FA is widely distributed in the plant world, where it occurs both in free form and bound to cell wall polysaccharides, lignin, glycoproteins, or polyamines. The richest natural sources are cereal bran (wheat, rice, corn), seed husks, as well as, in smaller amounts, certain herbs (including *Angelica sinensis*, *Cimicifuga racemosa*, and *Ligusticum chuangxiong*) and vegetables (tomatoes, carrots, corn) [37,38]. In industrial practice, FA is mainly obtained from agricultural and agro-industrial residues, such as by-products of beer production (brewer’s spent grain), using chemical processes (alkaline or acid hydrolysis) or, increasingly, enzymatic methods [39,40,41]. Enzymatic hydrolysis with the use of feruloyl esterases, carboxylesterases, or cholesterol esterases enables efficient and environmentally friendly release of FA from biomass, while enzyme additives such as xylanase can further increase process efficiency—in studies, the application of enzymatic pretreatment increased it from about 2.5–6 mg/g to 11.79 mg/g, i.e., nearly twofold or even fourfold compared to previous methods [37,41,42].

In addition to extraction from biomass, FA can be obtained through chemical and biotechnological synthesis. Chemical synthesis includes, among others, condensation reactions involving suitable phenolic precursors, but biotechnological methods, including metabolic engineering of microorganisms such as Escherichia coli, are gaining increasing importance. For example, by modifying biosynthetic pathways and optimizing enzyme activities (e.g., methyltransferases, hydroxylases), it is possible to achieve high yields of FA in fermentation on media from inexpensive raw materials such as wheat bran, glucose, starch, or glycerol [43,44,45]. In addition, strategies for cofactor regeneration (e.g., S-adenosylmethionine, flavin adenine dinucleotide) are being developed, which significantly increase the efficiency of biosynthesis [44].

Polyferulans, i.e., polymers of FA, can be obtained both through natural polymerization processes in plants and through controlled chemical or enzymatic polymerization in vitro. Oxidases, peroxidases, or suitable chemical catalysts are used for this purpose, allowing for the production of materials with diverse properties and potential applications in biomaterials and functional polymers [37,38].

## 3. Methods of Synthesis and Modification

### Synthesis of Polymers Based on Ferulic Acid and Derivatives

**Figure 3:**
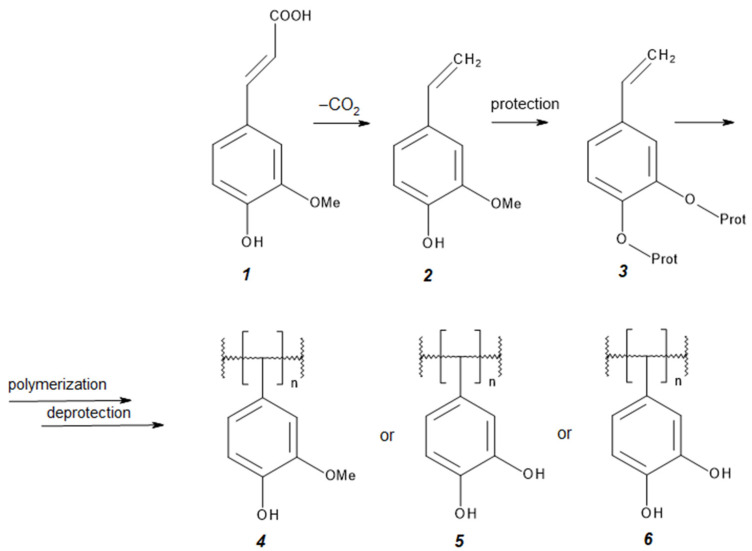
Scheme of the reaction for obtaining poly(vinylguaiacol) and poly(vinylcatechol). Prot—protective group (e.g., Si(OEt)_3_); 1—ferulic acid (FA); 2—4-vinylguaiacol; 3—protected bio-based functional styrene; 4–6—FA-derived polymers (adapted from [31]).

Strategies for the functionalization of polymers with FA include various approaches such as polymerization, grafting (attachment of side chains), and enzymatic modifications. Polymerization of FA derivatives, e.g., 4-vinylguaiacol, enables the production of well-defined styrene polymers such as poly(vinylguaiacol) or poly(vinylcatechol), while maintaining phenolic functionalities. This is achieved through controlled anionic or radical polymerization as well as appropriate protection and deprotection reactions of functional groups [26,31]. The synthesis route of these polymers is illustrated in Figure 3.

**Figure 4:**
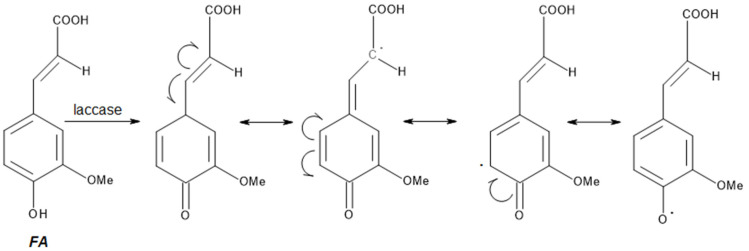
Formation of free radicals in oxidation of FA with laccase (adapted from [49]).

**Figure 5:**
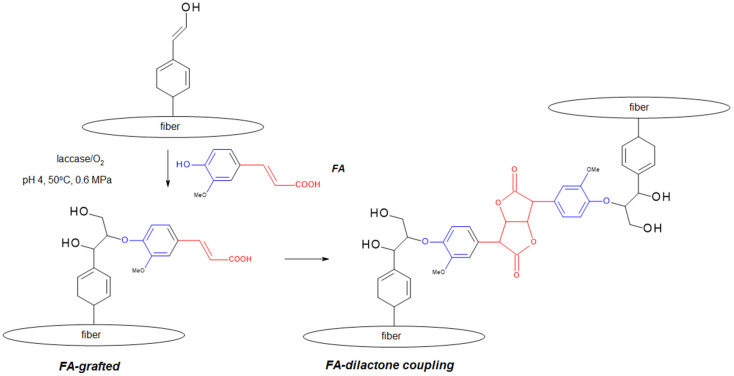
Example of FA modification of cellulose fibers—lactonization, 36% yield (adapted from [50]).

Grafting allows precise modification of polymer surfaces, e.g., polysaccharides or starch nanoparticles, through the attachment of synthetic polymer chains, which improves the physicochemical properties and bioactivity of materials [46,47,48]. FA can be oxidized by laccase, leading to the formation of highly reactive free radicals (Figure 4) [49]. Enzymatic modifications, using, among others, laccase from *Agaricus bisporus*, enable the attachment of oxidized FA products to biopolymers such as chitosan (CTS), carboxymethylcellulose, or CTS–gelatin hybrids, resulting in materials with enhanced antioxidant and antibacterial activity [26]. There are also reports in the literature on the use of this approach in laccase-assisted modification of lignocellulosic materials FA (Figure 5) [50].

Among the examples of polymers functionalized with FA are polyesters, which can be obtained through direct copolymerization of FA with diols, yielding biodegradable materials with UV-protective and hydrophilic properties. FA-based polyanhydrides exhibit controlled release of active substances and are attractive for biomedical and cosmetic applications [25]. Styrene polymers, such as poly(vinylguaiacol) or styrene copolymers, are obtained through both controlled polymerization and grafting methods, allowing precise tuning of their properties for specific applications [30,31,51]. Nanocomposites in which FA or its derivatives are incorporated into polymer matrices or onto nanoparticle surfaces exhibit improved mechanical properties, stability, and bioactivity, making them promising materials for applications in packaging, medicine, and environmental engineering, e.g., in water purification processes from heavy metals and other contaminants [46,47,48,52].

The literature describes many different types of polymers obtained from FA and its derivatives, which differ both in synthesis methods and in properties and potential applications. To better illustrate this diversity, Table 1 summarizes the most important examples of FA-based polymeric materials, including their methods of preparation, key functional features, and main application areas.

Table 1 summarizes the most important types of polymers obtained from FA and its derivatives, their synthesis methods, key properties, and main application areas. The above examples demonstrate that it is possible to obtain materials with diverse characteristics, such as biodegradability, antioxidant activity, barrier properties, or controlled release of active substances. The use of FA in polymers allows for the design of modern functional materials, which find applications in both biomedical fields and in the packaging industry or environmental engineering. In the following subsections, selected groups of materials and their application potential are discussed in detail.

## 4. Functional Properties and Applications

### 4.1. Physicochemical Properties of Ferulic Acid: Stability, Release, Antioxidant Activity, Mechanical Properties

FA and its derivatives exhibit diverse physicochemical properties that are crucial for their applications in polymers. The thermal stability of FA is limited, especially during high-temperature processing (decomposition temperature 206–245 °C [56]); however, chemical modifications, such as conjugation with polymers (e.g., CTS or poly(anhydride-esters)), significantly improve its resistance to degradation and enable controlled release of the active substance without loss of its biological properties [26,54,55]. Studies on polymer films have shown that FA derivatives can be released in a controlled manner, with the amount and rate depending on the type of polymer matrix and environmental conditions [26,54,57].

FA and its oligomers are strong antioxidants, capable of neutralizing free radicals through phenoxyl radical stabilization, and their antioxidant activity can even increase after dimerization or conjugation with polymers such as CTS [55,58,59,60]. Incorporation of FA derivatives, such as BDF, into polymers significantly improves the mechanical properties of the materials—for example, elongation at break increased from 5.2% for neat PLA to 431% for 40%-FA-blended PLA, depending on the concentration of the additive and the type of polymer used [35].

Furthermore, the presence of FA conjugated with CTS in polymer matrices can influence barrier properties, e.g., increasing the oxygen permeability resistance of films (0.1–20.6%) as measured according to ASTM D3985 at 25 °C and 0% relative humidity, with this effect rising with higher FA-CTS content [56]. Solubility, polymorphism, and compatibility with other components also play a role in designing functional polymeric materials incorporating FA [61,62].

### 4.2. Applications of Ferulic Acid

#### 4.2.1. Materials for Biomedical Applications (e.g., Drug Carriers, Skin Protection)

Polymers containing FA or polyferulic acid show great potential in biomedical applications, particularly as drug carriers and components of skin protection formulations [28,34,36,54,63,64,65]. Thanks to controlled biodegradation and the ability to gradually release FA, such systems enable long-lasting antioxidant and anti-inflammatory effects, which is especially valuable in wound healing, cancer therapy, and protection against oxidative stress induced by UV radiation [25,34,54,63,66]. Nanoparticles and nanofibers based on polyferulic acid can effectively deliver anticancer drugs, such as doxorubicin or paclitaxel, increasing their efficacy and limiting systemic toxicity, while themselves exhibiting anticancer activity [34,63]. Polymerized forms of FA are also used to create biocompatible coatings and films that improve the surface properties of biomaterials, reduce protein adsorption, and support tissue regeneration [33,64]. In skin care formulations, polymers containing FA provide stable and controlled release of the active substance, protecting skin cells from oxidative damage and photoaging [27,54,66]. Additionally, these systems demonstrate good biocompatibility and low cytotoxicity, as confirmed by in vitro and in vivo studies [54,63,64].

#### 4.2.2. Food Packaging, Biodegradable Materials, UV-Protective Films

Polymers containing FA or its derivatives are intensively studied as modern materials for food packaging due to their biodegradability, antioxidant and antibacterial properties, and ability to provide UV protection [67,68,69,70,71,72]. The addition of 1% FA to biopolymers, such as PLA or fish gelatin, significantly improves their mechanical, barrier, and thermal properties: the tensile strength of films made of PLA and poly(butylene adipate-co-terephthalate) increased from 5.42 MPa to 10.78 MPa, while ultraviolet (UV) transmittance at 280 nm decreased from 2.37% to approximately 1.7%, which reduces photodegradation and extends the shelf life of packaged food [68,70,72]. Active films and laminates containing FA exhibit strong antioxidant and antibacterial properties, limiting the growth of pathogens such as *Listeria monocytogenes*, *Escherichia coli*, and the fungus *Aspergillus niger*, while not compromising the sensory qualities of the packaged food [67,68,69,70]. Studies have reported, for example, composites made of polyethylene glycol and carboxymethylcellulose, in which the water vapor transmission rate decreased from approximately 1.8 × 10^−10^ to 1.1 × 10^−10^ g m^−1^ s^−1^ Pa^−1^ after the incorporation of FA and natamycin, additionally protecting food from drying and microbial growth [69]. Recently Huang and co-workers showed that synthetic lignin-like UV-blocking polymers can be obtained from FA [73].

#### 4.2.3. Adhesives

FA has attracted increasing interest for applications in modern adhesives and coatings, especially as a component of bio-based adhesive systems. Studies on photo-curable coatings based on methacrylates have shown that increasing the FA content significantly improves adhesion to glass and coating hardness. Specifically, pull-off adhesion measured according to [74] using Ø20 mm aluminum dollies ranged from 4.2 MPa to 5.5 MPa depending on FA content. Pendulum hardness, determined with a König pendulum according to [75], ranged from 53 a.u. to 79 a.u., with the optimal value observed for coatings containing 0.9 wt % FA.

The gloss (20° incidence) and distinctness of image (DOI) of the coatings, measured on white paper panels according to [76], were high, with gloss values of approximately 85–89 Gloss units (GU).

The antibacterial activity of the FA-modified coatings was assessed against Staphylococcus epidermidis following [77], showing a reduction of up to 3.8 log colony forming units (CFU)/m^2^ after 24 h [72].

In another study, FA-based epoxy adhesives showed excellent mechanical strength—for a 10 parts per hundred resin (phr) addition of hyperbranched FA epoxy resin, tensile strength measured according to [78] reached 126.4 MPa, with over 88.3% of that strength retained after recycling. Additionally, the stress relaxation time was only 45 s at 140 °C, facilitating processing and material reuse [79].

FA is also used to modify bio-based epoxy adhesives and photo-curable systems, improving not only mechanical properties but also enabling material recycling without loss of key parameters. Studies on FA-based epoxy monomers showed that appropriate curing strategies (thermal curing strategies—two-step curing at 150 °C for 2 h followed by 180 °C for 2 h) allow for materials with enhanced mechanical and adhesive properties. Tensile testing (ASTM D638) on 5B type dog-bone samples revealed a Young’s modulus of 774 MPa and a strain at break of 4.0%, while single lap shear tests [80] on aluminum, steel, and CMC composite substrates showed shear strengths of 20 ± 1 MPa, 24 ± 4 MPa, and 32 ± 3 MPa, respectively, with cohesive failure observed for the CMC joints and adhesive failure at the interface for metal substrates [81]. It is also worth noting that in soy-oil-based adhesives, FA modification led to changes in the failure mechanism (cohesive failure within the adhesive layer), indicating a strong influence of this compound on the adhesive structure and properties [82].

One of the main limitations is the low thermal stability of FA itself (decomposition temperature 206–245 °C [56]), which leads to its degradation during typical processing operations such as extrusion or injection molding. Studies have shown that pure FA has poor thermal stability, whereas its derivatives, such as BDF, exhibit significantly better retention (86–93%) during processing in PLA, polyhydroxybutyrate, and polyhydroxybutyrate-co-hydroxyvalerate matrices [28]. In PLA-based elastomers with FA derivatives, a significant increase in flexibility was observed—elongation at break increased from 6% for pure PLA to 434% for BDF modifications This enhancement, measured on tensile specimens prepared according to [83] is attributed to a physically connected network of interactions between PLA chains (PLA–PLA), between PLA and BDF (PLA–BDF), and between BDF molecules themselves (BDF–BDF). Such a network maintains the continuity of the amorphous phase in the polymer blend, allowing it to transition from brittle, plastic-like behavior to elastomeric behavior. The formation of this physical network, together with partial phase separation at higher BDF contents (>25 wt%), explains the dramatic increase in elongation at break from 6% for pure PLA to 434% for BDF-modified blends, as the network provides both mobility and cohesion under tensile stress [35]. The presence of FA can also affect the thermal properties of polymers—for example, lowering the glass transition temperature (Tg). In systems where FA has been chemically modified to form monoallylated monomers and used in thiol-ene photopolymerization, the addition of more monoallylated monomer leads to a decrease in the Tg of the crosslinked network. The mechanism of this effect is related to a decrease in the network density and a reduction in the restriction of polymer chain movement, which increases the mobility of network segments and acts similarly to plasticization [84]. In high-performance FA bio-epoxies, Tg can reach up to 250 °C, and their mechanical properties match or exceed those of traditional bisphenol A-based systems [85]. Modern fully bio-based epoxy vitrimer systems derived from FA exhibit excellent recyclability and mechanical stability. Mechanical tests conducted in accordance with ASTM D638 showed that the original FA-based epoxy vitrimer had a tensile strength of 126.4 ± 1.6 MPa, retaining 121.3 ± 3.1 MPa and 113.4 ± 2.1 MPa after the first and second reprocessing cycles—96% and 89%, respectively, of the initial strength. Even after chemical recycling, the vitrimer maintained over 88% of its original tensile strength [79].

## 5. Challenges and Future Perspectives

### 5.1. Limitations of Current Ferulic Acid Solutions

Current applications of FA and polyferulic acid in polymers face several significant limitations. Primarily, FA has low water solubility and limited bioavailability, which hinders its effective use in polymer systems, particularly in biomedical and pharmaceutical applications [36]. In PLA- and PHA-based polymers, FA release into polar environments is very slow (range of 8–15% of the content present in the film), limiting its functionality in food packaging or active materials [28]. The synthesis and functionalization of polyferulic structures often encounter performance barriers, as well as difficulties in controlling the degree of crosslinking and the availability of functional groups, affecting the mechanical and functional properties of the resulting materials. Additionally, limited enzymatic and steric accessibility of FA in polysaccharide matrices complicates efficient crosslinking and modification of mechanical properties [86]. Although the articles do not provide detailed financial data, the described enzymatic processes require the use of enzymes, indicating limitations in scalability and potential challenges for industrial applications [37,41]. Chemical synthesis methods of FA and its polymers allow for the production of larger quantities of products but require careful control of reaction conditions and the use of chemical reagents [30,31]. Finally, some FA degradation products may be difficult to fully mineralize in aqueous environments, posing challenges for recycling and biodegradation processes [87]. These limitations highlight the need for further research to improve stability, bioavailability, and the efficiency of synthesis, functionalization, and design of new polymers incorporating FA and its derivatives [28,36,86,87]. In the context of adhesives and thermoplastic polymers, the use of FA and its derivatives opens up new perspectives but also involves a series of technological challenges.

FA and its derivatives exhibit a very rapid rate of biodegradation in both aquatic and soil environments. Under laboratory conditions, in the presence of Paraburkholderia aromaticivorans bacteria, 75% of FA decomposes within 3 days (at temperatures ranging from 10 to 30 °C), and complete disappearance is observed after 6–9 days [88]. In soil, with the participation of the fungus Phomopsis liquidambari, more than 97% of FA is degraded within 48 h [89]. Under anaerobic conditions, FA is broken down into methane and carbon dioxide by bacterial consortia, although the duration of this process has not been precisely determined [90]. The incorporation of FA into biopolymers such as Poly(3-hydroxybutyrate-co-3-hydroxyvalerate) (PHBV) accelerates their biodegradation—in seawater, the half-life (t½) of PHBV films containing FA is 63–79 days, and complete biodegradation occurs within 162 days. For comparison, PLA under similar conditions shows a t½ of approximately 50–100 days, and PHA/PHBV 60–80 days [91]. This indicates that FA and its derivatives degrade faster or at rates comparable to typical biopolymers, which are considered benchmarks for biodegradability.

The degradation mechanisms of FA include hydrolysis of ester and anhydride bonds (in the case of polymers), as well as a series of enzymatic reactions: decarboxylation (FA decarboxylase), oxidation (laccase, peroxidase), and demethylation, leading to the formation of compounds such as vanillin and protocatechuic acid [89,92,93]. Microorganisms (bacteria and fungi) and environmental conditions play a key role in these processes—optimal pH (6–7) and the presence of enzymes significantly accelerate the degradation [88,89,92,93]. Under aerobic conditions, these processes occur much faster than under anaerobic ones, where FA undergoes mineralization to methane and CO_2_ [90].

### 5.2. Directions for Future Research and Potential New Applications of Ferulic Acid

Future research on FA and polyferulic acid in polymers should focus on the development of new materials for pharmaceutical applications. Among others, these are materials for advanced delivery systems, such as nanoformulations and nanocapsules, which increase bioavailability, stability, and controlled release of the active substance, especially in pharmaceutical and biomedical applications [64,94,95]. New FA derivatives, e.g., conjugated with amino acids, are being developed, exhibiting improved solubility, skin permeability, and potential for cosmetology and dermatology [96].

Further promising research areas involve several promising areas. One of these is FA-based vitrimers, used for the production of recyclable thermosetting materials. Studies on bio-based epoxies and hyperbranched FA systems have demonstrated the possibility of closed-loop recycling while retaining over 88% of mechanical strength [78,79], with the current Technology Readiness Level (TRL) estimated at 4–5, corresponding to laboratory validation and pilot-scale testing. Another area is nanocarriers for central nervous system drug delivery. FA in the form of nanoparticles and polyferulic nanocapsules enables controlled release of active substances, enhancing bioavailability and potential application in the treatment of neurological diseases such as Alzheimer’s and Parkinson’s [34,63,97,98]. The current TRL for this application is 3–4, covering in vitro and preclinical studies. A third promising direction is smart packaging with controlled release. Polymers containing FA exhibit antioxidant, antibacterial, and UV-protective properties, which can extend the shelf life of food products [37,67,68,69,70], with the current TRL estimated at 5–6, including pilot-scale production and packaging trials. Focusing research on these areas will allow more efficient exploitation of FA’s potential in polymeric materials and accelerate its implementation in industrial and clinical applications.

Potential applications also include treatment of neurological diseases, where FA, due to its ability to cross the blood–brain barrier and neuroprotective properties, could be used in therapies for disorders such as Alzheimer’s or Parkinson’s disease [97,98]. Promising directions also include smart packaging materials, which, due to the antioxidant and antibacterial properties of FA, can extend food shelf life [37].

Further research should include optimization of enzymatic and chemical synthesis, efficiency improvements, and sustainable scaled-up production from biomass [99]. It is also essential to deepen knowledge about safety, toxicity, and regulatory aspects of new formulations, especially for clinical applications [64,94]. Finally, the development of functional polymers with FA may find applications in tissue engineering, controlled drug delivery systems, anti-aging materials, and environmental protection [36,64,95].

In the context of adhesives and thermoplastic polymers, future studies should focus on improving the thermal resistance and stability of FA derivatives during processing, as well as optimizing their compatibility with polymer matrices. A natural development direction is the use of FA in thermoplastic materials that can serve as polymer matrices or hot-melt adhesives. Developing new derivatives, crosslinking strategies, and active substance delivery systems could significantly expand FA applications in adhesives and thermoplastics, particularly for functional, biodegradable, and recyclable materials.

The following SWOT analysis (Table 2) summarizes the strengths and weaknesses, as well as the opportunities and risks associated with the use of FA and its polymers in polymeric materials.

To sum up SWOT, FA polymers have significant market potential due to their functional properties and renewable origin. Limitations include difficulties in scaling enzymatic processes and challenges in controlling the properties of materials. Chemical synthesis methods offer the possibility of larger-scale production, which can facilitate industrial implementation, although they require careful control of the reaction conditions.

### 5.3. Toxicity, Safety, and Regulatory Aspects

FA exhibits a favorable safety profile in in vitro and in vivo studies, although its toxicity depends on dose and application method. Tests on cell lines and animal models have shown that FA at moderate concentrations does not produce significant cytotoxic effects and, on the contrary, protects cells exposed to environmental toxins such as deoxynivalenol, lead, cadmium, or arsenic, mitigating oxidative stress, inflammation, and apoptosis [102,103,104,105]. However, at high concentrations, FA can induce toxic effects, including hemolysis of erythrocytes and negative impacts on intestinal wall integrity, suggesting the need for dose control in supplements and food products [100,101].

In silico and in vitro studies confirm low human toxicity of FA, but potential environmental toxicity has been reported, which should be considered when designing biodegradable polymer materials [100]. In pharmaceutical and biomedical applications, growing attention is being paid to nanoformulations, which can improve the safety and effectiveness of FA delivery while minimizing adverse effects [94].

In studies to date, oral LD_50_ for FA in rats has been found to be approximately 2113 mg/kg body weight for females and 2445 mg/kg body weight for males [98]. However, long-term chronic toxicity data for FA and its polymers remain limited, particularly regarding repeated-dose exposure, reproductive toxicity, and environmental impact of FA-containing polymers. In silico predictions also suggest low acute toxicity (LD_50_ ≈ 1.43 mol/kg for rats) but do not replace long-term safety evaluations.

The registration report by the European Chemical Agency (ECHA) for 4-hydroxy-3-methoxycinnamic acid as a registered substance indicates that in the 4-week oral test in F344 rats, dietary administration of 2% FA (equivalent to a nominal dose of 2000 mg/kg/day) did not cause observable adverse effects, suggesting that the NOAEL is at least 2000 mg/kg/day [106]. However, additional studies covering chronic and reproductive endpoints are needed, especially for FA when incorporated into polymeric materials, as polymer conjugation may alter release kinetics, bioavailability, and tissue distribution.

From a regulatory perspective, FA meets pharmacokinetic criteria (compliance with Lipinski’s rule), supporting its further development as a component of drugs, supplements, and biomedical materials [67]. Nevertheless, before widespread implementation, additional toxicological studies, long-term safety assessments, and compliance with standards for food-contact materials and clinical applications are required [94,100,101]. Further research should address potential changes in toxicity resulting from polymer conjugation, nanoparticle formation, and controlled-release systems while establishing safety margins and exposure limits.

## Figures and Tables

**Table 1:** Examples of polymers based on FA and its derivatives.

Derivative Type	Synthesis/Modification Method	Key Properties	Main Applications	Source
Ferulate polyesters	Copolymerization of FA with diols	Biodegradable; UV-protective; hydrophilic; elongation at break 6–434% (PLA-based, Bis-O-dihydroferuloyl-1,4-butanediol (BDF)-modified)	Packaging, biomaterials, cosmetics	[25,28,35]
Ferulate polyanhydrides	Anhydride polymerization	Controlled release of active substances, biodegradability	Biomedical applications, cosmetics, drug delivery systems	[25,53]
Styrene polymers	Anionic/radical polymerization, protection of groups	Preservation of phenolic groups, tunable properties	Functional materials, elastomers	[30,31,34,51]
FA-based nanocomposites	Grafting, enzymatic modifications, incorporation	Improved mechanical properties (Tensile strength increased from 5.42 to 10.78 MPa), stability, bioactivity, antibacterial activity	Packaging, environmental engineering, biomaterials	[46,47,48,52,54]
FA-modified CTS polymers	Enzymatic modifications (e.g., laccase)	Enhanced antioxidant and antibacterial activity, biocompatibility, oxygen permeability resistance 0.1–20.6%	Biomaterials, coatings, protective films	[26,33,55]
Polyferulans (FA polymers)	Chemical, enzymatic, natural polymerization	Biodegradability, biological activity, potential for controlled release	Biomedical applications, packaging, drug delivery systems	[36,37,38]

**Table 2:** SWOT analysis for FA.

Strengths	Weaknesses
Renewable feedstocks (lignocellulose, plant residues) [23,24] Biodegradability and antioxidant activity [25,28] Potential for polymer functionalization (grafting, enzymatic modification) [46,47,48,49,50]	Low water solubility of FA [36] Challenges in controlling crosslinking degree and availability of functional groups [100] Limited scalability of some enzymatic processes [37,41]
**Opportunities**	**Threats**
Applications in pharmacy and biomaterials (controlled release, nano-systems) [34,36,64] Biodegradable and UV-protective packaging materials [67,68,69,70,71,72] Modification of adhesives and photo-curable resins [72,73,79,81,82]	Challenges in scaling enzymatic processes to industrial production [37,41] Competition from chemical and petrochemical polymers [30,31] Regulatory challenges and safety requirements for pharmaceutical and food-contact applications [94,100,101]

## Data Availability

No new data were created or analyzed in this study. Data sharing is not applicable to this article.

## Abbreviations

The following abbreviations are used in this manuscript:

BDF	Bis-O-dihydroferuloyl-1,4-butanediol
CFU	Colony forming units
CTS	Chitosan
DOI	Distinctness of Image
ECHA	European Chemical Agency
FA	Ferulic acid
GU	Gloss units
IUPAC	International Union of Pure and Applied Chemistry
PHA	Polyhydroxyalkanoates
PHBV	Poly(3-hydroxybutyrate-co-3-hydroxyvalerate)
phr	Parts per hundred resin
PLA	Polylactic acid
Tg	Glass transition temperature
TRL	Technology Readiness Level
